# An Image Signal Accumulation Multi-Collection-Gate Image Sensor Operating at 25 Mfps with 32 × 32 Pixels and 1220 In-Pixel Frame Memory

**DOI:** 10.3390/s18093112

**Published:** 2018-09-15

**Authors:** Vu Truong Son Dao, Nguyen Ngo, Anh Quang Nguyen, Kazuhiro Morimoto, Kazuhiro Shimonomura, Paul Goetschalckx, Luc Haspeslagh, Piet De Moor, Kohsei Takehara, Takeharu Goji Etoh

**Affiliations:** 1 Department of Industrial and Systems Engineering, International University, Vietnam National University HCMC, Linh Trung Ward, Thu Duc District, Ho Chi Minh City, Vietnam; dvtruongson@gmail.com; 2 School of Science and Engineering, Ritsumeikan University, 1-1-1 Noji-Higashi, Kusatsu, Shiga 525-8577, Japan; 18v00169@gst.ritsumei.ac.jp (N.N.); rr0056fp@ed.ritsumei.ac.jp (K.M.); skazu@fc.ritsumei.ac.jp (K.S.); 3 School of Electronics and Telecommunications, Hanoi University of Science and Technology, 1 Dai Co Viet Road, Ha Noi, Vietnam; quang.nguyenanh@hust.edu.vn; 4 IMEC, Kapeldreef 75, B-3001 Leuven, Belgium; Paul.Goetschalckx@imec.be (P.G.); Luc.Haspeslagh@imec.be (L.H.); Piet.DeMoor@imec.be (P.D.M.); 5 School of Science and Engineering, Kindai University, 3-4-1 Kowakae, Higashiosaka City, Osaka 577-8502, Japan; takehara@civileng.kindai.ac.jp

**Keywords:** high-speed, image sensor, ISAS, image signal accumulation, MCG

## Abstract

The paper presents an ultra-high-speed image sensor for motion pictures of reproducible events emitting very weak light. The sensor is backside-illuminated. Each pixel is equipped with the multiple collection gates (MCG) at the center of the front side. Each collection gate is connected to an in-pixel large memory unit, which can accumulate image signals captured by repetitive imaging. The combination of the backside illumination, image signal accumulation, and slow readout from the in-pixel signal storage after an image capturing operation offers a very high sensitivity. Pipeline signal transfer from the MCG to the in-pixel memory units enables the sensor to achieve a large frame count and a very high frame rate at the same time. A test sensor was fabricated with a pixel count of 32 × 32 pixels. Each pixel is equipped with four collection gates, each connected to a memory unit with 305 elements; thus, with a total frame count of 1220 (305 × 4) frames. The test camera achieved 25 Mfps, while the sensor was designed to operate at 50 Mfps.

## 1. Introduction

### 1.1. Fusion of Image Signal Accumulation and Multiple Collection Gate Image Sensors

**Figure 1:**
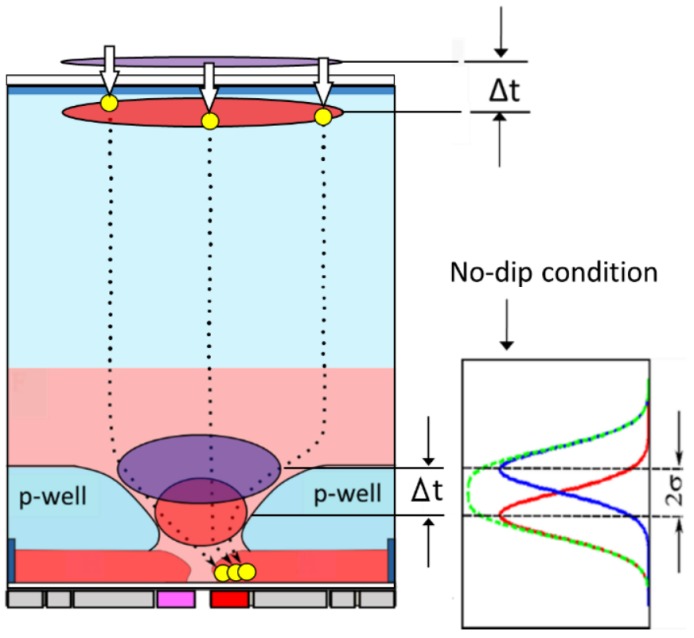
A cross-section of the backside illuminated multi-collection-gate image signal accumulation sensor ([BSI MCG ISAS] the temporal resolution limit Δt = 2σ, where σ is the standard deviation of the arrival time of the signal electrons to one of the collection gates).

**Figure 2:**
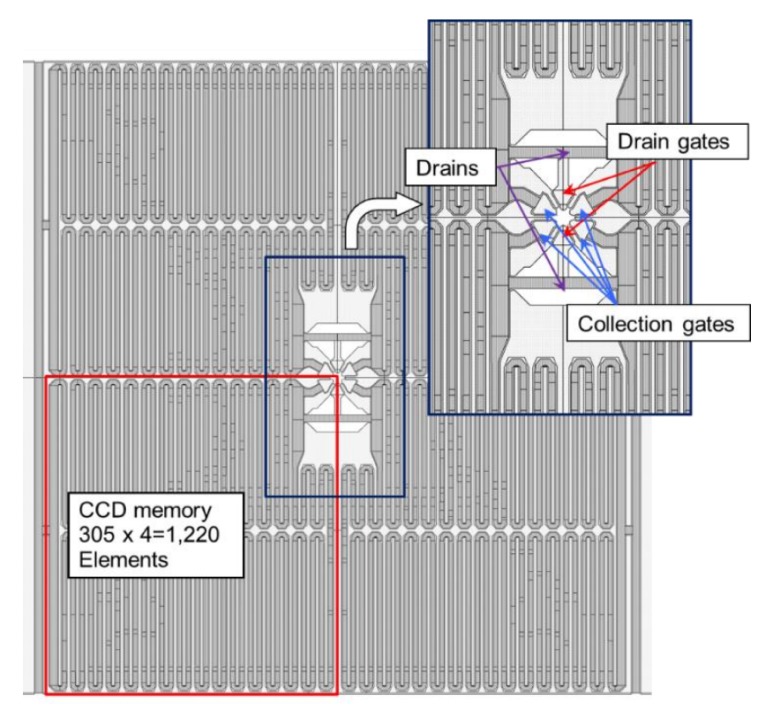
A pixel of the BSI MCG ISAS with four collection gates and four in-pixel folded looped CCD memories (the frame count is 1220 frames).

The paper presents a small test sensor, named BSI MCG ISAS, using two sensor concepts, the image signal accumulation sensor (ISAS) [1] and the backside-illuminated multi-collection-gate image sensor (BSI MCG) [2]. Figure 1 conceptually depicts a cross-section of the pixel. Figure 2 shows the real pixel structure of the test sensor.

The BSI MCG image sensor operates at an ultrahigh frame rate, keeping a high sensitivity by backside illumination. They have four to eight charge collection gates spreading like flower petals at the center of the front side of each pixel. A high driving voltage (VH), 1.5 to 3 volts higher than a low driving voltage (VL), is applied to one of the collection gates, which collects the signal charges generated by the incident light in the backside layer. The voltage VH is applied to the collection gates, in turn, at very short intervals. The charges generated by rapidly varying incident light are separately collected at the intervals to form consecutive signal charge packets. One disadvantage of the BSI MCG image sensor is a small frame count, equal to the number of collection gates.

The ISAS is capable of the in-pixel accumulation of a large number of consecutive image signals, captured by the repetitive imaging of reproducible events. Weak image signals provided by ultra-high-speed imaging are enhanced by signal accumulation without being read out of the pixel. As shown in Figure 2, one CCD memory is placed in each quadrant of a pixel. The CCD memory is looped with the first element connected to the last element. For example, the image signals captured in the second image capture operation are added to those captured in the first operation, and are stored in the in-pixel CCD memory.

The BSI MCG ISAS enables the ultra-high-speed imaging of reproducible phenomena emitting very weak light and a replay of the motion picture with the large frame count, which is advantageous in their applications to imaging TOF-MS, pulse-neutron tomography, and imaging of signal propagation on the brain surface.

### 1.2. Pipeline Operation by Four Collection Gates and In-Pixel Four-Phase CCD Memories

The pipeline operation of the BSI MCG ISAS combining the ISAS and the BSI MCG image sensor enables both the large pixel count and the very large frame rate. The test sensor shown in Figure 2 has two drain gates and four collection gates at the center of each pixel.

**Figure 3:**
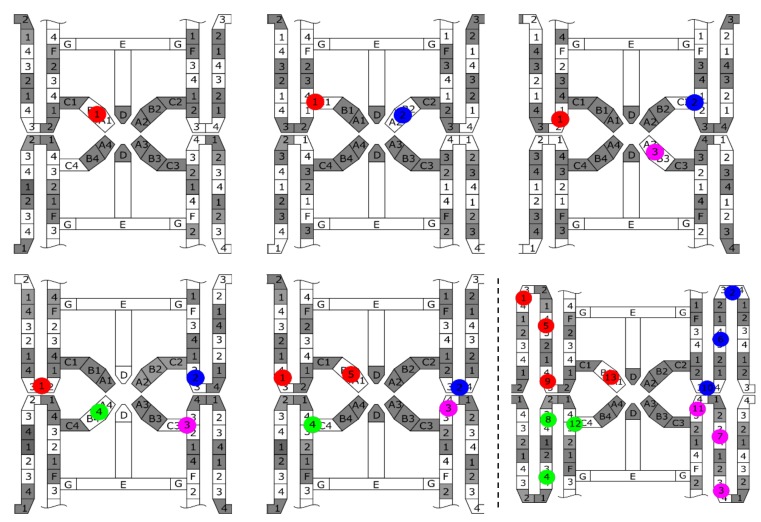
The pipeline operation of the combination of four collection gates and four-phase CCDs.

Each of the collection gates is connected to a looped four-phase CCD memory with 305 elements, providing the total frame count of 1220 frames (4 × 305). The four-phase CCD operation provides a perfect pipeline operation with the four collection gates, as shown in Figure 3. The charge packet shown with a red circle and numbered 1 is collected by the collection gate 1 (A1), and is transferred to the first element of the CCD memory attached to the collection gate A1. During the transfer, the remaining three collection gates, A2 to A4, collect the charge packets in turn. Then, the collection gate A1 is empty and can receive the fifth charge packet.

## 2. Design of Test Sensor

### 2.1. Specifications

The specifications of the test chip of the BSI MCG ISAS are shown in Table 1. The pixel count is only 32 × 32 pixels. The pixel size is as large as 72.56 μm so as to have a large CCD memory in each pixel. Although the frame rate in the design was 50 Mfps, the test camera worked at 25 Mfps. The sensor was cooled down to −40 °C to reduce the large dark current. The active area of the pixel is as small as 2.32 mm (32 pixels × 72.56 μm). The reasons for the lower frame rate and the large dark current of the test sensor are explained in Section 3.4.

### 2.2. P-Well Design and Frame Rate Evaluation

The temporal resolution of the camera is determined by the temporal resolution of the sensor chip and the performance of the driver. The temporal resolution of the sensor chip is defined by 2σ of the arrival time of the signal electrons to one of the collection gates, where σ is the standard deviation, as shown in Figure 1 [3]. The potential profile is designed to decrease the standard deviation of the arrival time of the electrons. Empirically, however, the standard deviation is highly correlated with the average arrival time. Therefore, the p-well masks and the implantation energy of the Boron ions are deigned to decrease the average arrival time. The lower field makes the drift velocity lower and the diffusion coefficient higher, both decreasing the arrival time. Therefore, the minimum field along the pass of the electrons should be maximized. The solution is a linear potential or a constant field. This strategy was first suggested by the authors [4] in the design of the image sensor that achieved the frame rate of 1 Mfps for the first time in the world. Since then, the design principle has been applied to designing every element of the CCD channels and the photo-diode of our sensors [1,2].

**Figure 4:**
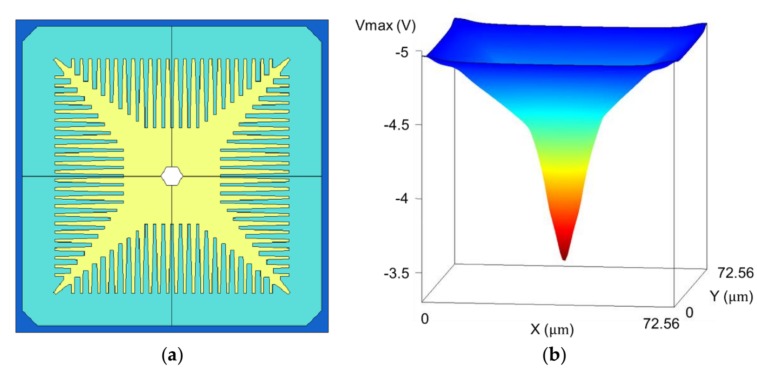
Three masks to create the p-well and the resultant potential profile (superposed). (**a**) The following three masks to create the linear p-well potential: mask 1 (yellow) covering the whole area except the center hole, mask 2 (light blue) with the comb-like structure, and mask 3 (blue) like a frame along the pixel boundary. The implantation energies are also different. (**b**) The potential profile over the p-well made with the masks (the linear potential is created, except the area near the center and the boundary of the pixel).

**Figure 5:**
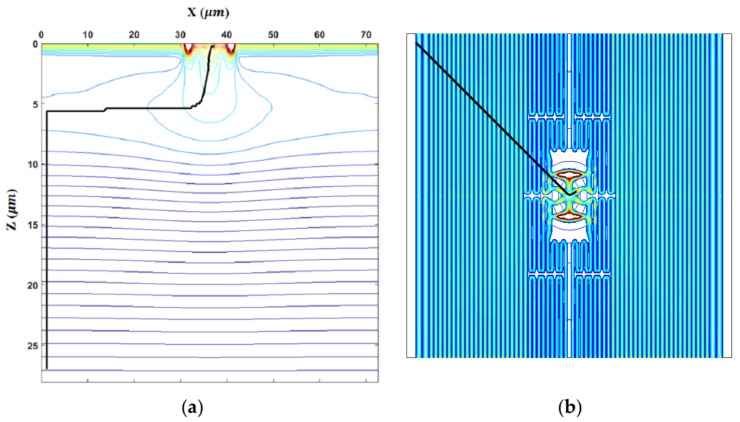
A cross-section of the potential profile created by the p-well and a trajectory of an electron without the random motion: (**a**) cross section; (**b**) on the *x*–*y* plane (bends are simplified, except in the central area, to avoid excessive calculation time).

The three masks for the p-well of the test sensor and the resultant potential profile are shown in Figure 4. The potential profile with an example trajectory of an electron is shown in Figure 5.

The design guide of the p-well masks was as follows:

(1) p-well mask 1 (yellow): The first mask for the p-well covers the whole pixel area, except the center hole. The implantation energy is low.
(2) p-well mask 2 (light blue): The second mask is designed like combs, with many straight twigs narrowing toward the tip from the four edges at the pixel boundaries.
(3) p-well mask 3 (blue): The third mask simply covers the pixel boundary. The implantation energy was chosen at the practically highest value of the process provided by the foundry.

The width of the third mask is the minimum value required for the highest energy. As the implantation energy for the mask is very high, the mask should be thick enough to prevent the Boron ions from penetrating. As the mask shape is trapezoidal with the sloping edges, the thick mask increases the minimum width of the mask. On the other hand, the mask must be as narrow as possible, as the wide p-well mask 3 results in a wide low-field area near the boundary, causing spatial crosstalk due to the electrons crossing the boundary, and the lower temporal resolution due to the signal electrons arriving very late at the collection gate from the boundary area.

As shown in Figure 4b, the design method effectively linearized the potential profile, except the areas very close to the pixel boundary and the central area. The milder slope near the pixel boundary is due to the wide p-well 3 masks. The steep potential in the central area is due to the transient potential to the center hole to introduce the signal electrons to the front side. Actually, it is not so acute. The slope in the middle area is extremely low, about 0.02 V/μm. To make it visible, the slope in the central area is exaggerated.

**Figure 6:**
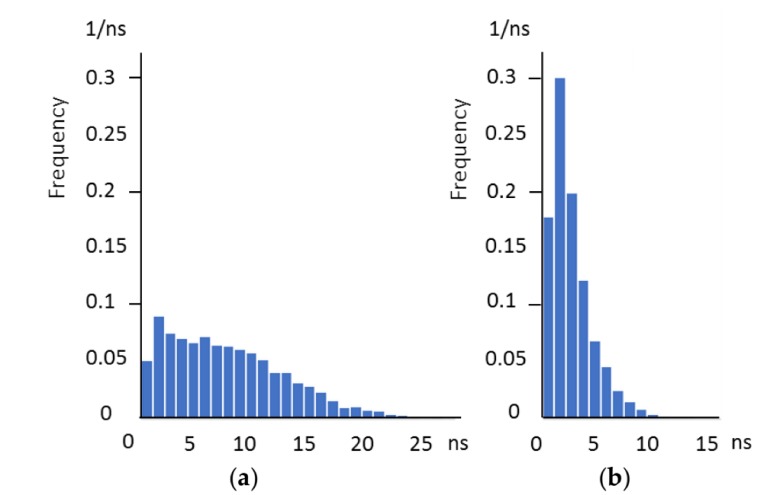
The arrival time distribution of signal electrons: (**a**) 100% fill factor and (**b**) 25% fill factor.

The temporal resolution is evaluated by the Monte Carlo simulation. Figure 6 shows the frequency distribution of the arrival time of electrons to the collection gate for fill factors of 100% and 25%. Table 2 shows the average, the standard deviation σ, the temporal resolution defined by the no-dip condition for the Gaussian distribution, Δt = 2σ, and the 95%, t_95_, for the fill factors of 100%, 25%, and 10%.

As the ratio of the 95% and the no-dip condition falls in a small range, 1.62 to 1.70, either of the definition of the temporal resolution can be converted to the other one by multiplying the average of the factor 1.66. If the frame interval is set to the 95%, the temporal crosstalk can be practically neglected. Therefore, the 95% was used to decide the frame rate of the sensor in the design.

(1) For the 100% fill factor, the frame interval of 18.1 ns (55.2 Mfps) can be achieved. Therefore, the design frame rate is set at 50 Mfps. Then, the design operation rate of the four collection gates and the in-pixel CCD memories are both 12.5 Mfps.
(2) For the 10% fill factor, the frame interval of 2.75 ns (364 Mfps) can be achieved. The collection gates can be operated by a driver circuit on the driver chip stacked to the sensor chip.

### 2.3. Folded Looped In-Pixel CCD Memory

Although the folded CCD in-pixel memory was proposed in early 1990s [5], it was impossible to fabricate the structure at the time. In 1996, Kosonocky made a breakthrough by introducing the serial-parallel-serial (SPS) CCD for the in-pixel memory [6]. However, the yield rate was very low. In 2001, Etoh and Mutoh proposed a practical in-pixel CCD memory structure, using a linear CCD memory slightly slanted to the pixel grid, to avoid the bends in the folded CCD and the right-angle signal transfer from a serial CCD to parallel CCDs in the SPS CCD. The sensor with the slanted linear CCD memories achieved 1 Mfps for the first time [4].

**Figure 7:**
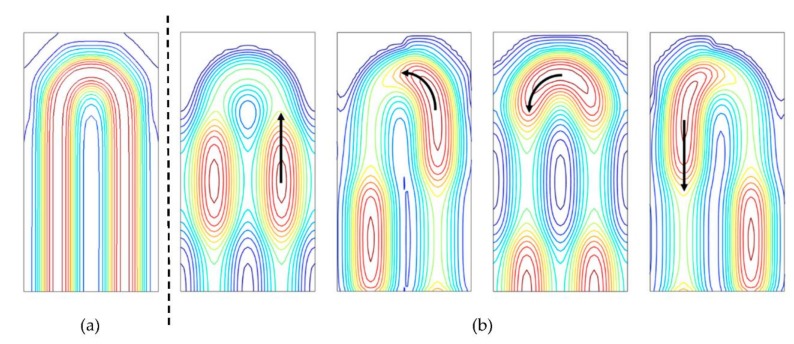
Potential profiles at a bend (**a**) in the design, the same high driving voltage (VH) is applied to the bend area without the electrode gaps, and the channel potential is flattened with the allowance of 0.05 V difference. (**b**) Changes in the electrodes with the high voltage VH in turn transport the signal charge packets.

The current fine mono-layer electrode CCD process enables full curvilinear design. However, the curvilinear design with a large number of bends in a pixel takes a very long time. The design of each bend differs depending on the acceptor concentration at the front side to create the p-well, with different potentials at different positions in a pixel. Furthermore, the straight channel elements and the bends were designed to maintain the same potential, with an allowance of 0.05 V across the entire pixel. It was achieved, as shown in Figure 7a, yet with insufficient considerations regarding the local high field appearing at the bends in Figure 7b.

The fine mono-layer electrode CCD process also revived the SPS CCD in-pixel memory for ultra-high-speed imaging [7].

## 3. Evaluation of Test Sensor

### 3.1. Pipe-Line Operation of Four Collection Gates and Four-Phase CCD

**Figure 8:**
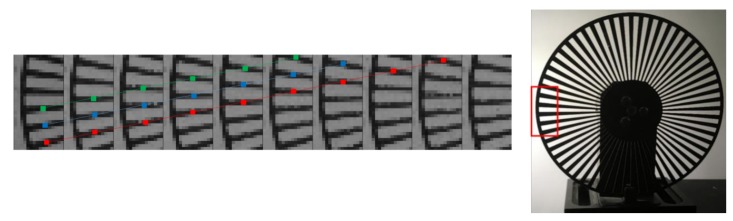
Confirmation of the pipeline signal transfer by the combination of four collection gates and four-phase CCD memories (the images were taken at 100 kfps; every 15 frames were selected, and stitched to show the smooth motion; the photo on the right side is a reference taken by a different camera).

To confirm the pipeline operation of the combination of four collection gates and four-phase transfer CCDs, images of a rotating laser beam chopper were captured at a slow speed at 100 Kfps. The images of every 15 frames taken from the consecutive frames are shown in Figure 8. The zigzag images of the frames are due to the 32 pixels rows. Careful observation confirmed that no strange images, such as duplicated or missing images, appears.

### 3.2. Frame Rate

**Figure 9:**
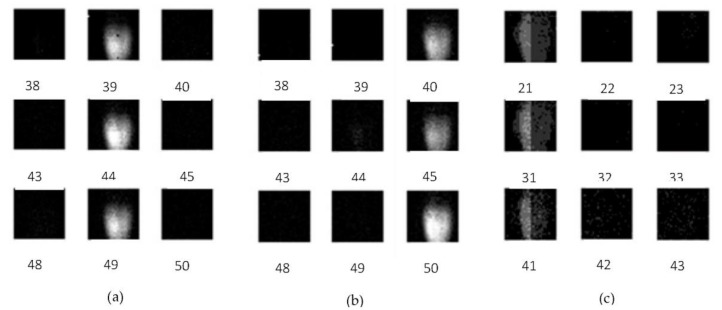
The laser diode (LD) images (the LD directly illuminates the sensor through a very dark ND filter). (**a**) Every five frames for 12.5 Mfps; (**b**) every five frames for 25 Mfps; (**c**) and every 10 frames for 50 Mfps.

Figure 9 shows the images of a laser diode (LD) taken by the test camera with the test sensor. The total pulse width of the LD was less than 400 ps, and the beam diameter is about 1 mm. The small active area of about 2.32 mm (32 pixels × 72.56 μm) is directly irradiated with the laser pulses through an ND filter to prevent damage to the sensor.

The LD illuminated every five frames for the image capturing at 12.5 Mfps and 25 Mfps, and every ten frames for 50 Mfps, due to the limited repetition rate of the pulse generator in our laboratory with a sufficient power to control the LD.

The LD images captured at 12.5 Mfps are clear and have no afterimage; those at 25 Mfps show slight instability; and those at 50 Mfps are weak, and the left and the right halves show a strong contrast, but no after image.

### 3.3. Image Signal Accumulation

**Figure 10:**
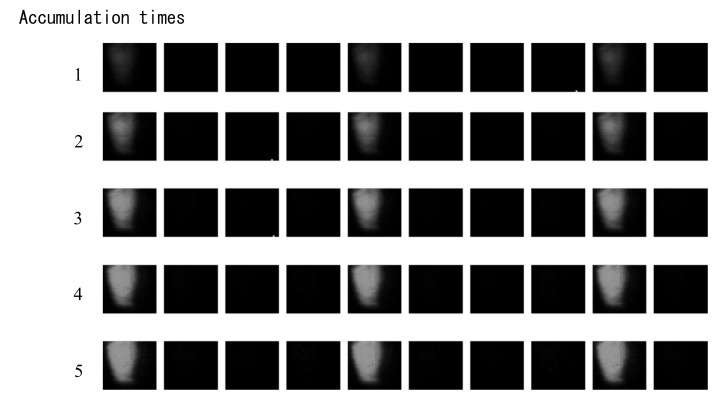
Confirmation of signal accumulation (captured at 12.5 Mfps; the LD is applied every four frames; only very weak images appear for the usual first image capture, and they become clearer with increase of the accumulation times; no afterimage appears even after five-time accumulation with 1525 [5 × 305] transfer steps on each looped CCD, proving a reasonable transfer efficiency at least up to 12.5 Mfps).

Figure 10 shows the images taken to confirm the image signal accumulation. The sensor was irradiated with very weak LD pulses for every four frames at 12.5 Mfps. The images in the first row were taken in the first image capturing operation and read out; those in the second row were captured by accumulation in a continuous two-round image capture without intermission. The experiments were repeated five times for accumulation.

Only very faint images appear in the first row, and are enhanced with the accumulation of the signals by the repetitive image captures.

### 3.4. Trouble Shooting of Major Problems

#### 3.4.1. Maximum Frame Rate of the Sensor

The highest frame rate of a camera is limited by the performance of the sensor and the driver on the driver board. The evaluation revealed that the waveform of the driving pulses deformed at the operation rate higher than 25 Mfps. A Monte Carlo simulation of the motion of signal electrons, shown in Figure 6a and Table 2, indicates that the practically perfect charge collection is possible at the interval of 20 ns (50 Mfps). Therefore, the main cause of the lower frame rate may be the insufficient performance of the driver of the test camera. To overcome the problem, a three-dimensional (3D)-stacking of the driver chip is planned for the prototype.

#### 3.4.2. Large Dark Current

The cause of the large dark current was identified to be the very high local field. The curvilinear design is used for the folded memory CCD channels and the CCD bridges from the collection gates to the memory CCDs. Our limited design skill and the technical difficulty for the curvilinear design in a limited design period caused the local high field. The design technology has been improved, and is now being used to modify the design of the prototype sensor.

## 4. Future BSI MCG ISAS

The signal accumulation is also possible with in-pixel CMOS memories and a CMOS image accumulation circuit [8].

The 3D-stacking enables the stacking of a driver chip with in situ driver circuits almost free from the RC delay due to wiring, which significantly multiplies the frame rate.

The 3D-stacking technology enables the installment of the in situ AD converter and in situ digital memories in the stacked chip. However, the signal recording in the in-pixel analogue memories is much faster than in the in-pixel digital memories. On the other hand, once the signals are stored in the in-pixel or near-pixel digital memories and are slowly read out, dark current and readout noise can be small and practically neglected. Therefore, the ideal ultra-high-speed image sensor in the near future is equipped with both an in-pixel analogue memory and an in situ digital memory, with the digital memory elements per pixel equal to or more than the in-pixel analogue memory elements. Immediately after the ultra-high-speed image capture with the analogue memory, the stored analogue signals are slowly transferred to the in situ digital memory through the in situ AD convertors, each of which converts the analogue signals of a group of the pixels to the digital signals [9].

## 5. Concluding Remarks

A small ultra-high-speed image sensor with a special function for in-pixel image signal accumulation was developed and evaluated. The characteristic performance, the very high frame rate of 25 Mfps, and the signal accumulation functions were confirmed. Two major problems were identified, namely: the frame rate did not reach the design target 50 Mfps, and the dark current was very large. The causes were identified.

For the prototype sensor, the 3D-stacking of the driver chip over the sensor chip is planned. Then, the RC delay will no longer be a limited factor to achieve 50 Mfps. As shown in Figure 6 and Table 2, if a lower fill factor is allowed with an on-chip micro-lens array, the sensor chip can increase the frame rate to more than 100 Mfps. The in situ driver circuits on the stacked driver chip will support the achievement.

The design of the prototype is progressing, carefully avoiding any local high field in the complicated CCD channel design.

Suzuki et al., presented another test sensor that achieved the frame rate more than 50 Mfps and the frame count about frames [10]. These sensors will promote innovations in advanced measurement technologies requiring ultra-high speed, very high sensitivity, and long continuous imaging.

## Figures and Tables

**Table 1:** Specifications of the test backside-illuminated multi-collection-gate image signal accumulation sensor (BSI MCG ISAS).

Sensor Structure	Functional Backside Illumination
Maximum Frame Rate	25 Mfps (Target: 50 Mfps)
Pixel Count	32 × 32
Pixel Size	72.56 µm × 72.56 µm
Fill Factor	100%
Sensor Size	3.6 mm × 4 mm
Number of Collection Gates	4
Number of Consecutive Frames	1220 frames (305 memory elements/quadrant)
Size of CCD Elements	1 µm × 3.2 µm
Charge Handling Capacity	3000 electrons
Overwriting Drain	Installed
Transfer Scheme	four-phase transfer
Temperature of Sensor	−40 °C

**Table 2:** Parameters on the electron arrival time distribution.

Fill Factor	Mean	Standard Deviation σ	Temporal Resolution (Δt = 2σ)	95% (t_95_)	t_95_/Δt
100%	7.81 ns	5.51 ns	11.02 ns	18.1 ns	1.64
25%	2.62 ns	1.99 ns	3.98 ns	6.60 ns	1.66
10%	1.31 ns	0.81 ns	1.62 ns	2.75 ns	1.70
